# Supplementary material for: Topologically protected elastic waves in phononic metamaterials
Source: Nat Commun. 2015 Nov 4;6:8682. doi: 10.1038/ncomms9682 (PMC4659837; doi:10.1038/ncomms9682)
Supplement: Supplementary Information — Supplementary Figures 1-9, Supplementary Table 1, Supplementary Notes 1-6 and Supplementary References [file ncomms9682-s1.pdf]

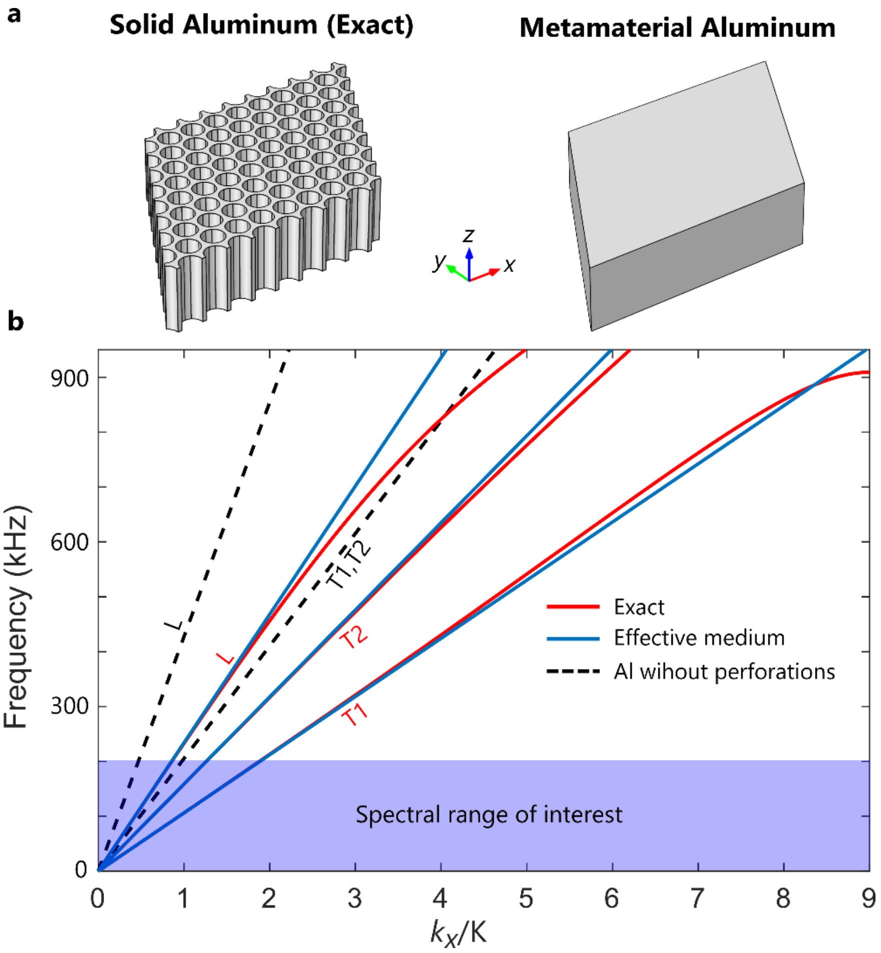

Supplementary Figure 1 | Bulk elastic modes of solid and metamaterial aluminum. (a) Three different geometries are studied: (1) perforated treated as is, (2) perforated treated as an effective medium, and (3) solid aluminum. Perforations are of the form of a triangular lattice of air holes. Radius of the holes is 0.0423 that of the crystal period. The period of the perforations to that of the lattice is 1/9 (Thus the filling factor of the air holes is 0.649). The effective metamaterial aluminum is simulated as an unperforated aluminum slab with effective elastic properties as given in Supplementary Table 1. (b) Dispersion diagram of the low-frequency modes of all three structures along the X direction. k_y and k_z are assumed to be zero. Red curves correspond to the exact solution and the blue curves correspond to the approximate simulation using the effective elastic moduli given in Supplementary Table 1. The spectral range of interest (<200 kHz) shows excellent match between the two approaches. Dashed black curves show the dispersion without any perforations, in which the two transverse modes, T1 and T2, are degenerate. In the perforated material, however, these transverse modes are no longer degenerate. The longitudinal mode L is also modified by the perforations.

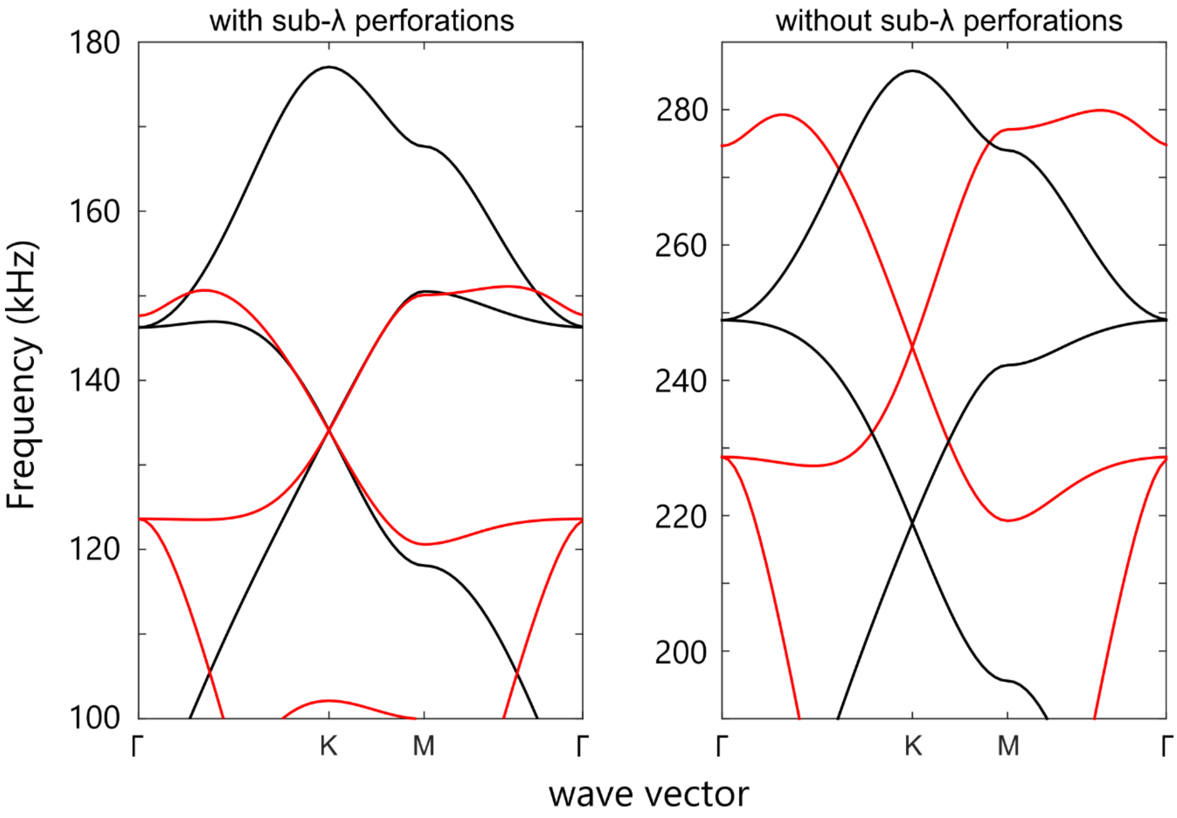

Supplementary Figure 2 | Dispersion diagram for the structure shown in Figure 1 of the main text, with and without the sub-wavelength perforations. Matching both the Dirac frequency and Dirac velocity is quite hard without the sub-wavelength perforations, shown in Supplementary Figure 1a.

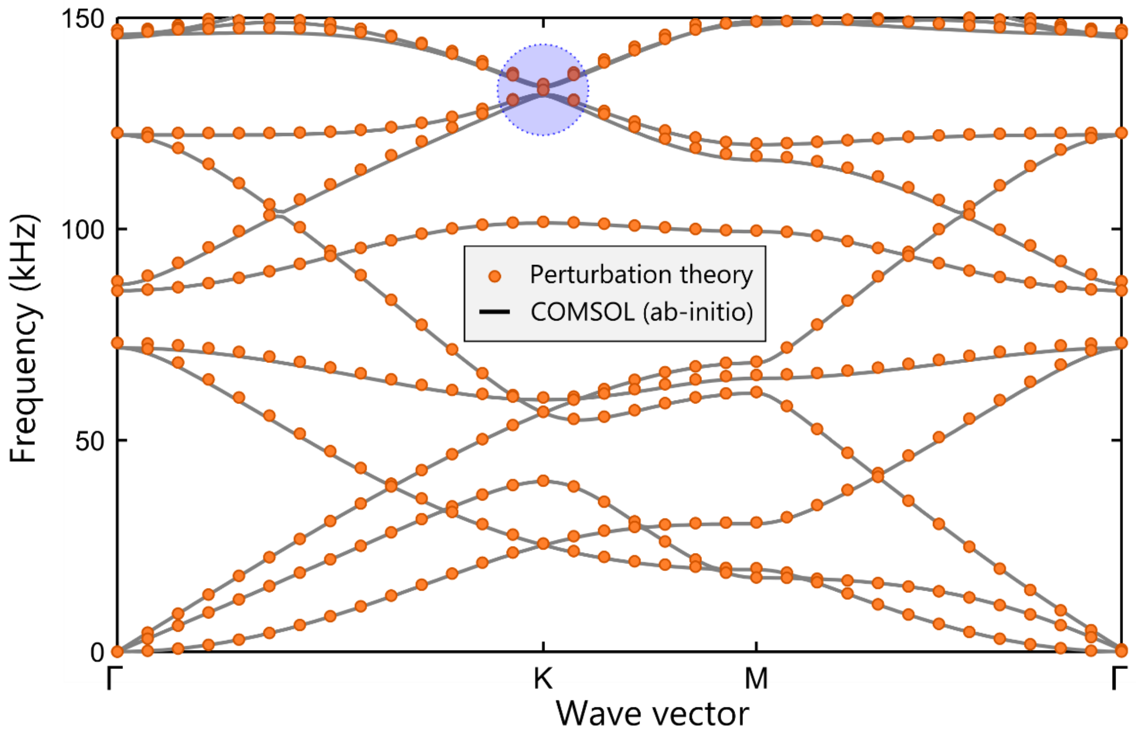

Supplementary Figure 3 | Band structure of the perturbed structure obtained from first-principles FEM simulation of the perturbed structure (solid lines) and first-order perturbation theory in conjugation with the unperturbed modes obtained from FEM simulations (markers). The topological bandgap marked by the blue circle (as well as the rest of the spectrum) is accurately predicted by the perturbation theory given in Supplementary Note 2.

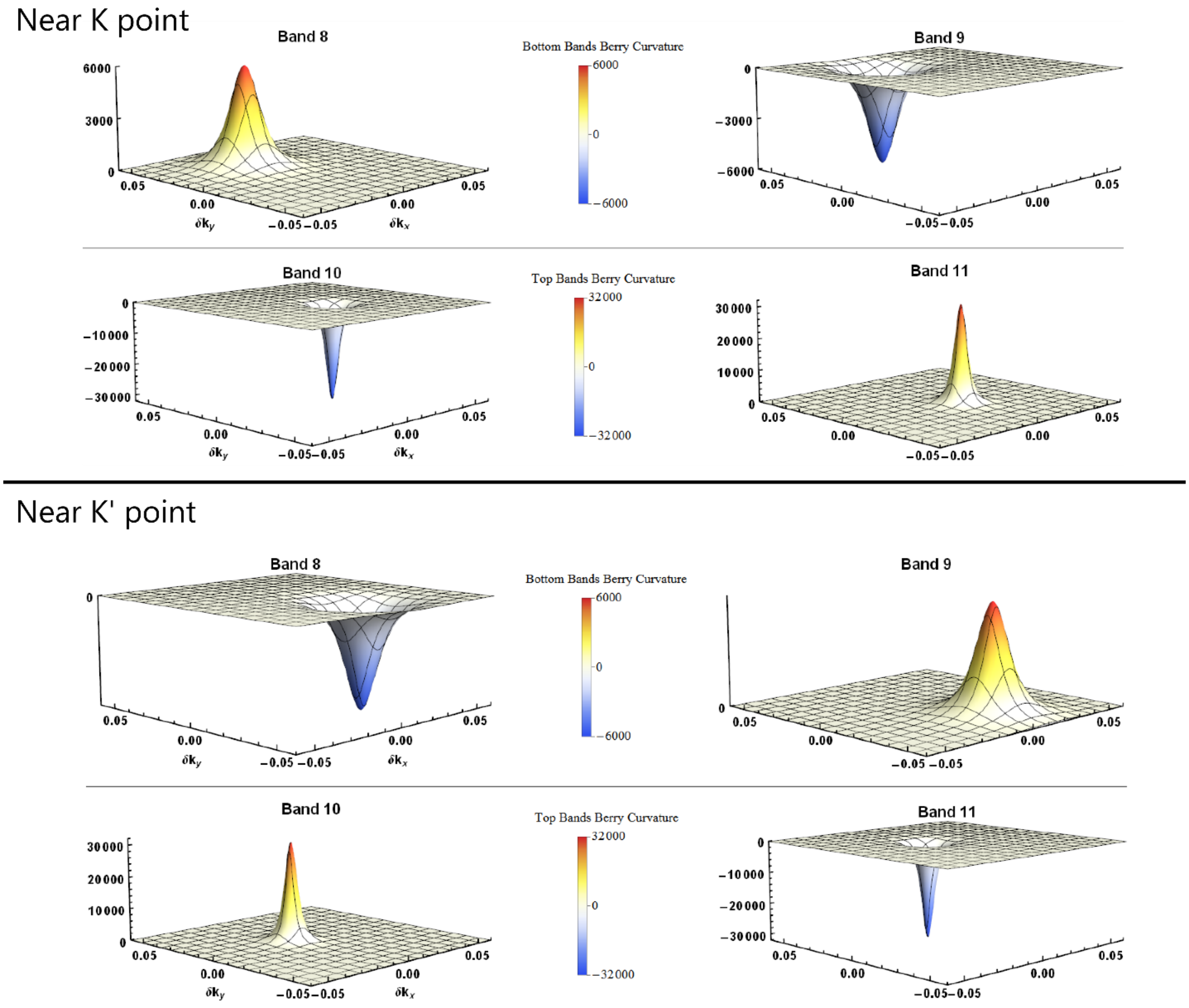

Supplementary Figure 4 | Berry curvature neat the K (top) and K' (bottom) points for four bands of interest. Berry curvature is numerically calculated from the perturbed structure eigenmodes (see Fig. 2 of the main text) using first-principles FEM COMSOL simulations. The area under each peak (dip) is equal to π ($-\pi$). Note that the degenerate bands have opposite sign of Berry curvature, characteristic of QSHE.

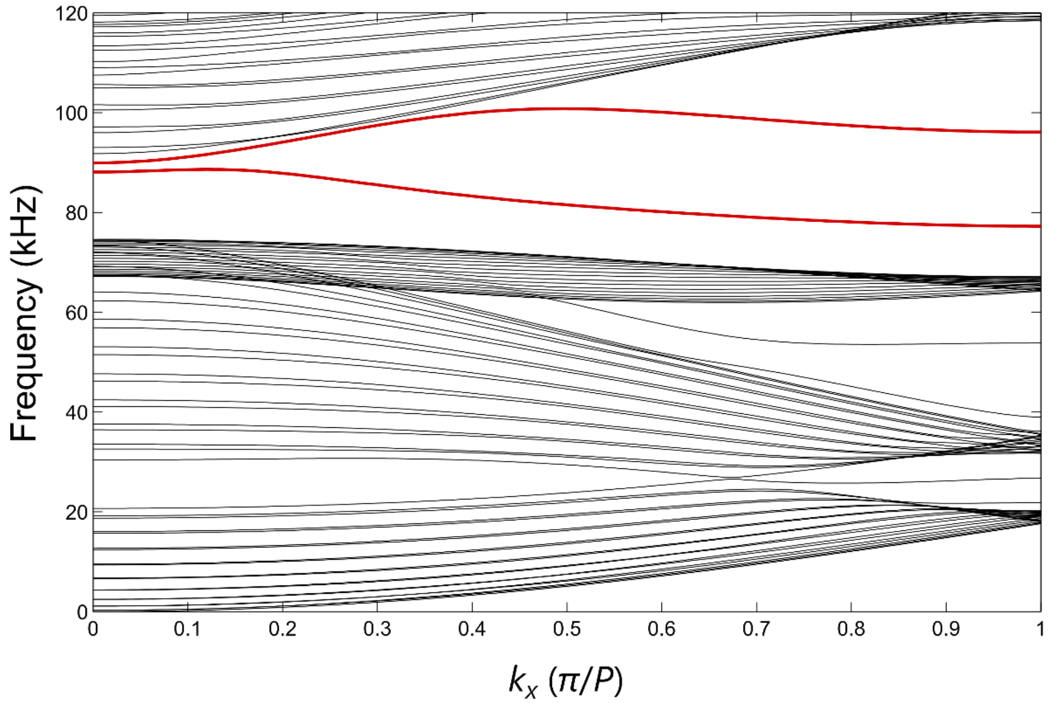

Supplementary Figure 5 | Bandstructure of the surface (red) and bulk (black) modes for the line defect given in Fig. 5a of the main text.

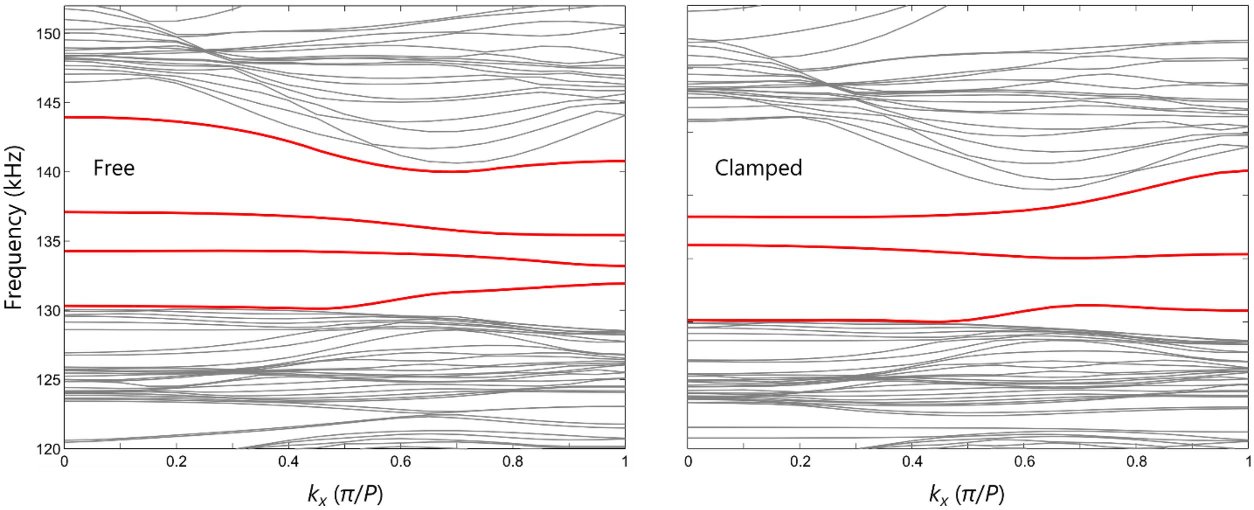

Supplementary Figure 6 | The free and clamped external boundaries support topologically trivial surface modes (shown by the red curves). Here, a supercell of 10x1 unit cells with free (left panel) and clamped (right panel) boundaries on both ends of the supercell was simulated.

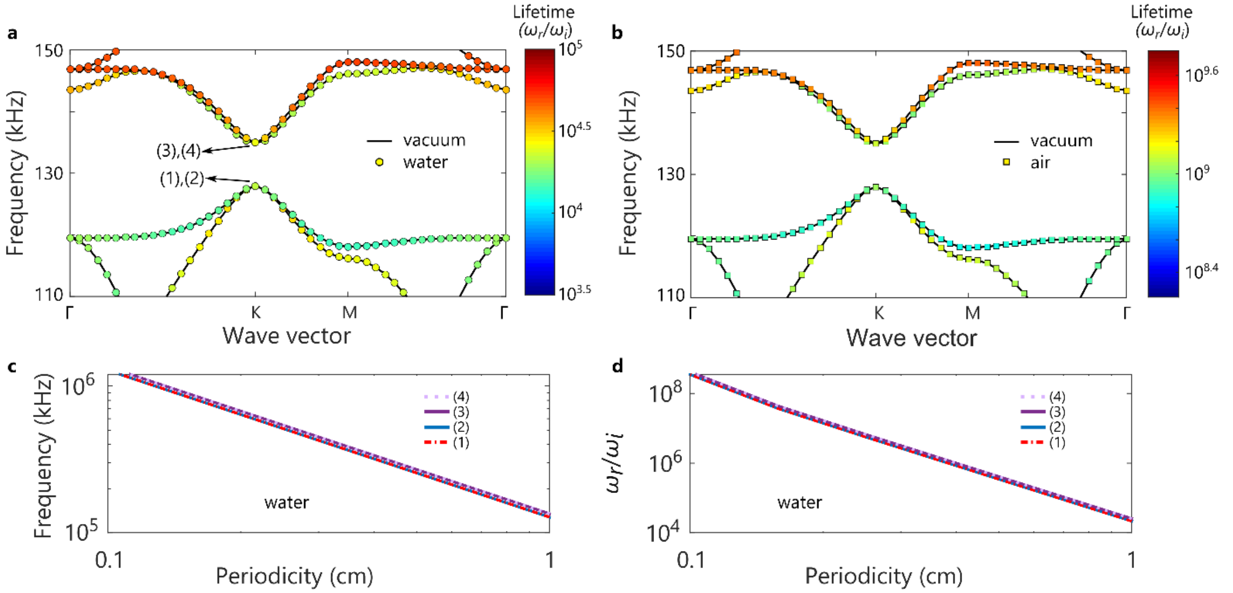

Supplementary Figure 7 | Modification of the modes lifetime due to the interaction with viscous fluids, water (a) and air (b), shown by markers. The case without a fluid (vacuum) is shown by black solid lines. Lifetime, defined as the ratio of the real part to the imaginary part of the eigenfrequency, is plotted by colour in log scale. (c,d) show how downscaling the structure proportionally to the periodicity increases both the frequencies (c) and lifetimes (d). Four frequency points (doubly degenerate modes at the K point, shown in a) are chosen as examples. In (c-d), the structure is assumed to be embedded in water. Other parameters are: c_{air}=343.2 m/s, c_{water}=1481.4 m/s, ρ_{air}=1.2 kg.m^{-3}, ρ_{water}=999.6 kg.m^{-3} .

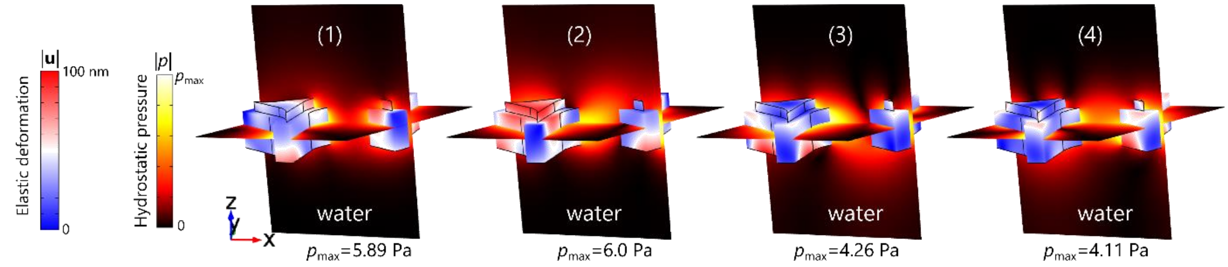

Supplementary Figure 8 | Hydrostatic pressure field profile generated in water due to the vibrating elastic crystal. Two cuts of the unit cell are shown: *z*=0 and *y*=0. The elastic deformations are assumed to be 100 nm in amplitude. The maximum generated pressure is denoted below each panel. The panels correspond to the same frequency points marked in Supplementary Figure 7 as (1) to (4). The simulations were performed in COMSOL Multiphysics using coupled 3D full-wave elastic and acoustic fields.

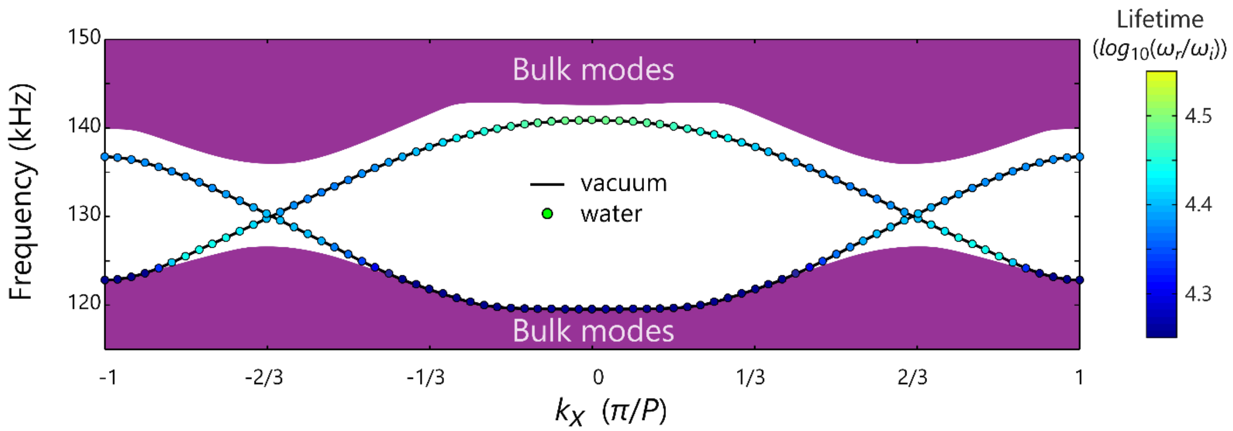

Supplementary Figure 9 | Effect of water loading on the topologically protected edge modes propagating between two domains with the counterbores made on opposite faces. Markers corresponds to the water environment and black lines are associated with the vacuum case. Attenuation is shown in colour in log scale.

Supplementary Table 1| Phononic Metamaterials Effective Stiffness Tensor

Stiffness tensor components and mass density of solid and metamaterial aluminum. The second column is extracted from fitting to the dispersion of low-frequency modes in Christoffel model.

Solid unperforated aluminum (isotropic)	Metamaterial aluminum (hexagonal)
$\boldsymbol{c_{11} = c_{33} = \lambda + 2\mu = 110.7\ \mathrm{GPa}}$	$c_{11} = 11.7$ GPa
$\boldsymbol{c_{12} = c_{13} = \lambda = 59.6\ \mathrm{GPa}}$	$c_{33} = 28.8$ GPa
$\boldsymbol{c_{44} = c_{66} = \mu = 25.6\ \mathrm{GPa}}$	$c_{12} = 6.9$ GPa
$\boldsymbol{\rho = 2700\ \mathrm{kg.m^{-3}}}$	$c_{13} = c_{23} = 6.5$ GPa
Assuming: Young's modulus E = 69 GPa	$c_{44} = c_{55} = 5.4$ GPa
and Poisson's ratio $\boldsymbol{\nu = 0.35}$	$\rho = 947.5\ \mathrm{kg.m^{-3}}$

Supplementary Note 1 | Effective Medium Theory

The metamaterial with sub-wavelength patterns used in this work was modeled as an effective uniform medium. This approximation holds very accurately as long as the size of the patterns remains much smaller than the wavelength of interest.

To find the effective elasticity parameters of the metamaterial, we studied the dispersion of very-low-frequency modes of the patterned structure. These modes are described by the Christoffel model of a continuum medium. According to this model, the dispersion of the modes are described by the Christoffel tensor $\mathbf{\Gamma}$:

$$\rho \left(\frac{\omega}{k}\right)^2 \vec{\mathbf{u}} = \mathbf{\Gamma}\,\vec{\mathbf{u}}\,, \tag{1}$$

where ρ is the continuum mass density, $\vec{\mathbf{u}}$ the displacement field, ω the frequency, and k the propagation constant, of the eigenmode. The Christoffel tensor $\mathbf{\Gamma}$ is related to the stiffness tensor and the propagation direction: $\Gamma_{il} = c_{ijsl}\frac{k_j k_s}{|k|^2}$, where all the indices run from 1 to 3. By calculating the modes dispersion for different propagation directions and finding the ratio of ω/k, it is possible to find the entire 'effective' stiffness tensor $\mathbf{c}$.

The crystal considered in this work possesses hexagonal symmetry. Such a crystal is described by five nontrivial elements of the stiffness matrix

$$\mathbf{c} = \begin{bmatrix} c_{11} & c_{12} & c_{13} & 0 & 0 & 0 \\ c_{12} & c_{11} & c_{13} & 0 & 0 & 0 \\ c_{13} & c_{13} & c_{33} & 0 & 0 & 0 \\ 0 & 0 & 0 & c_{44} & 0 & 0 \\ 0 & 0 & 0 & 0 & c_{44} & 0 \\ 0 & 0 & 0 & 0 & 0 & \frac{c_{11}-c_{12}}{2} \end{bmatrix} \tag{2}$$

Note that here we have used the Voigt representation[1] to reduce the number of indices. Supplementary Table 1 lists the nontrivial components of the stiffness tensor for both solid unperforated aluminum and an aluminum metamaterial made by sub-wavelength patterning as shown in Supplementary Figure 1a. Note the strong degree of anisotropy of the perforated structure by comparing the two columns of the table. This strong anisotropy was a key in achieving the degeneracy between the otherwise highly non-degenerate symmetric and anti-symmetric modes of an elastic slab.

Supplementary Figure 1b shows how well the results of the effective medium simulations match the exact solution in the frequency range of interest (<200 kHz). Even though the fitting parameters were obtained

by considering the very low frequency modes, as can be seen from the dispersion diagram the match works quite well even for high frequecnies. In very high frequencies (>200 kHz), the perforations start to play signicant role and some resonance features can be seen up in the dispersion diagram. However, as long as we are interested in the wavelengths as large as the unit cell size (here, 9 times the period of the sub-wavelength holes), no distinction may be drawn between the two approaches. This is why we confidently used the effective medium theory in solving the large-domain simulations, whose computation can be very demanding and frequenctly unfeasible.

Finally, in Supplementary Figure 2 we show that without the non-resonant sub-wavelength perforations, achieving the matching conditions for both the Dirac frequency and Dirac velocity are hard. The perforations are auxiliary knobs that result in additional anisotropy enabling such matching.

Supplementary Note 2 | First-Order Perturbation Theory

First-order perturbation theory can be used to explain the effects of a small perturbation to the eigenmodes of an elastic system. In our case, the system is perturbed by carving out some of the elastic material, resulting in a change of the resonator's free external boundary. The boundary with vacuum is characterized by zero traction $\hat{T} \cdot \boldsymbol{n} = 0$. Below we will explain how we can construct a new set of eigenmodes from the unperturbed ones such that they satisfy this new boundary condition at the shifted boundary.

Both unperturbed and perturbed eigenmodes satisfy the following Cauchy elastic equations:

$$\begin{gathered} \boldsymbol{\nabla}.\mathbf{T} = i\omega\rho\vec{\mathbf{v}} \\ \boldsymbol{\nabla}_s\vec{\mathbf{v}} = i\omega\mathbf{S} \end{gathered} \quad , \tag{3}$$

where $\mathbf{T}$ is the stress tensor, $\mathbf{S}$ the strain tensor, $\vec{\mathbf{v}}$ the velocity vector, ω the angular frequency, ρ the density, and $\boldsymbol{\nabla}_s = 1/2\ (\boldsymbol{\nabla} + \boldsymbol{\nabla}^T)$ is the symmetric gradient operator. The only difference between the perturbed and unperturbed cases is in the boundary conditions. To find the projection of the perturbed eigensolutions on the unperturbed ones, we multiply (from the left side) the first line of Supplementary Eq. 3 by $\vec{\mathbf{v}}_m^*$ and the second line by $\mathbf{T}_m^*$. Here, $\vec{\mathbf{v}}_m$ and $\mathbf{T}_m$ are the velocity and strain fields of the *m*-th unperturbed eigenmode. After integrating the result of these dot products over a volume V, we find the following projection equations:

$$\begin{gathered} \oint_S \vec{\mathbf{v}}_m^* \cdot \mathbf{T} \cdot \boldsymbol{n}\, da + i\omega_m \int_V \mathbf{T}_m^* \cdot \mathbf{S}\, dV = i\omega \int_V \vec{\mathbf{v}}_m^* \cdot \rho \cdot \vec{\mathbf{v}}\, dV \\ \oint_S \vec{\mathbf{v}} \cdot \mathbf{T}_m^* \cdot \boldsymbol{n}\, da + i\omega_m \int_V \vec{\mathbf{v}}_m^* \cdot \rho \cdot \vec{\mathbf{v}}\, dV = i\omega \int_V \mathbf{T}_m^* \cdot \mathbf{S}\, dV \end{gathered} \quad , \tag{4}$$

where S is the surface enclosing the volume V. Above, we used the vector identity $\vec{\boldsymbol{a}} \cdot \boldsymbol{\nabla} \cdot \mathbf{M} = -\boldsymbol{\nabla}_S \vec{\boldsymbol{a}} \cdot \mathbf{M} + \boldsymbol{\nabla} \cdot (\vec{\boldsymbol{a}} \cdot \mathbf{M})$. Let us assume that V is the volume of the perturbed structure. Therefore, everywhere on the surface S, the boundary condition of zero traction holds $\hat{T} \cdot \boldsymbol{n} = 0$. Note that this is only true for the perturbed solution, since the surface S is only a virtual boundary for the unperturbed system. Summing the two lines in Supplementary Eq. 4, we obtain the expression for the new eigenvalues:

$$i(\omega - \omega_m) = \frac{\oint_S \ \vec{\mathbf{v}} \cdot \mathbf{T}_m^* \cdot \boldsymbol{n} \, da}{\int_V \ [\mathbf{T}_m^* \cdot \mathbf{S} + \vec{\mathbf{v}}_m^* \cdot \rho \cdot \vec{\mathbf{v}}] \, dV} \tag{5}$$

The surface integral can be rewritten into a volume integral proportional to the small change in the volume ΔV. Reversing the sign of surface normal so that the volume enclosed by S is ΔV – the removed volume – and using the above-mentioned vector identity once again, one can rewrite the surface integral as a volume integral:

$$\oint_S \ \vec{\mathbf{v}} \cdot \mathbf{T}_m^* \cdot (-\boldsymbol{n}) \, da = \int_{\Delta V} \nabla \cdot [\vec{\mathbf{v}} \cdot \mathbf{T}_m^*] \, dV = i\omega \int_{\Delta V} \mathbf{T}_m^* \cdot \mathbf{S} \, dV - i\omega_m \int_{\Delta V} \vec{\mathbf{v}}_m^* \cdot \rho \cdot \vec{\mathbf{v}} \, dV$$
$$\approx i\omega_m \int_{\Delta V} [\mathbf{T}_m^* \cdot \mathbf{S} - \ \vec{\mathbf{v}}_m^* \cdot \rho \cdot \vec{\mathbf{v}}] dV \tag{6}$$

Combing Supplementary Eqs. 5 and 6, we reach at the final expression for the change in the eigenvalue due to the removal of the volume by ΔV:

$$\frac{\omega - \omega_m}{\omega_m} = \frac{\int_{\Delta V} [-\mathbf{T}_m^* \cdot \mathbf{S} + \vec{\mathbf{v}}_m^* \cdot \rho \cdot \vec{\mathbf{v}}] dV}{\int_V \ [\mathbf{T}_m^* \cdot \mathbf{S} + \vec{\mathbf{v}}_m^* \cdot \rho \cdot \vec{\mathbf{v}}] \, dV} \tag{7}$$

Supplementary Eq. 7 is closely related to the Slater theory of perturbed electromagnetic cavities.[2] Assuming that the perturbation is small, the new eigenfunctions can be expanded in terms of the unperturbed ones: $\vec{\mathbf{v}} = \sum \phi_n \vec{\mathbf{v}}_n$, and $\mathbf{T} = \sum \phi_n \mathbf{T}_n$. In this case, Supplementary Eq. 7 can be recast into an eigenvalue problem:

$$\sum\nolimits_n \omega_m (U_{mn} + \Delta_{mn}) \, \phi_n = \omega \sum\nolimits_n U_{mn} \phi_n \tag{8}$$

where $\Delta_{mn} = \int_{\Delta V} [-\mathbf{T}_m^* \cdot \mathbf{S}_n + \vec{\mathbf{v}}_m^* \cdot \rho \cdot \vec{\mathbf{v}}_n] dV$ and $U_{mn} = \int_V \ (\mathbf{T}_m^* \cdot \mathbf{S}_n + \vec{\mathbf{v}}_m^* \cdot \rho \cdot \vec{\mathbf{v}}_n) \, dV$. By properly choosing the arbitrary normalization of the unperturbed eigenmodes ($U_{mn} = \delta_{mn}$), one can further simplify the form of Supplementary Eq. 8.

Case of 4 Modes Representing Two Overlaid Dirac Bands

The first-order perturbation theory developed in the previous section can be used to treat degenerate cases as well. In the system considered in this paper, there are 2 overlaid Dirac cones, amounting to 4 degenerate modes at the K point (bands 8 to 11, as shown in Supplementary Figure 3) and quasi-degenerate in its vicinity due to having approximately the same group velocity. Here, in this section, we will develop a low-energy Hamiltonian describing the hybridization between these modes in the close vicinity of the K point. We assume that the linear dispersion of the modes is engineered such that they all have the same Dirac frequency (ω_D) and Dirac velocity (V_D), therefore, $\omega_i(\delta\mathbf{k}) = \omega_D \pm V_D|\delta\mathbf{k}|$. The upper and lower branches of the Dirac modes are labeled as (*I*) and (*II*), respectively, in the superscript. Two of the unperturbed modes are symmetric (here, labeled as S^I and S^{II}), and two are anti-symmetric (here, labeled as A^I and A^{II}). From the numerical simulations we observe that only the lower S and upper A as well as the lower A and upper S modes interact; all the other off-diagonal elements of the Δ_{mn} matrix are zero.

$$\begin{bmatrix} -V_D|\delta\boldsymbol{k}| + \omega_D\Delta_{S^IS^I} & 0 & 0 & \omega_D\Delta_{S^IA^{II}} \\ 0 & V_D|\delta\boldsymbol{k}| + \omega_D\Delta_{S^{II}S^{II}} & \omega_D\Delta_{S^{II}A^I} & 0 \\ 0 & \omega_D\Delta^*_{S^{II}A^I} & -V_D|\delta\boldsymbol{k}| + \omega_D\Delta_{A^IA^I} & 0 \\ \omega_D\Delta^*_{S^IA^{II}} & 0 & 0 & V_D|\delta\boldsymbol{k}| + \omega_D\Delta_{A^{II}A^{II}} \end{bmatrix} \begin{bmatrix} \phi_S^I \\ \phi_S^{II} \\ \phi_A^I \\ \phi_A^{II} \end{bmatrix} = (\omega - \omega_D) \begin{bmatrix} \phi_S^I \\ \phi_S^{II} \\ \phi_A^I \\ \phi_A^{II} \end{bmatrix} \quad (9)$$

In the main text we demonstrated that by adjusting the slab thickness, it is possible to achieve two sets of double degenerate eigenmodes with a bandgap in between such that the slope of the dispersion is zero (i.e., $\partial_k\omega = 0$) at the K point (i.e., when $\delta k = 0$). It is easy to see that such condition can be satisfied only if $\Delta_{S^IS^I} = \Delta_{S^{II}S^{II}} = \Delta_{A^IA^I} = \Delta_{S^{II}S^{II}} \equiv \Delta\omega/\omega_D$, and $|\Delta_{S^IA^{II}}| = |\Delta_{S^{II}A^I}|$. By redefining the arbitrary phase of the unperturbed eigenmodes, the off-diagonal elements of the $\boldsymbol{\Delta}$ matrix are made to take the form $\Delta_{S^IA^{II}} = -\Delta_{S^{II}A^I} \equiv im/\omega_D$, from which the 'mass' term m in band theory is defined. Taking all these considerations into account, the 'Hamiltonian' (Supplementary Eq. 9) can be written as

$$\begin{bmatrix} -V_D|\delta\mathbf{k}| & 0 & 0 & i\,m \\ 0 & V_D|\delta\mathbf{k}| & -i\,m & 0 \\ 0 & i\,m & -V_D|\delta\mathbf{k}| & 0 \\ -im & 0 & 0 & V_D|\delta\mathbf{k}| \end{bmatrix} \begin{bmatrix} \phi_S^I \\ \phi_S^{II} \\ \phi_A^I \\ \phi_A^{II} \end{bmatrix} = (\omega - \omega_D - \Delta\omega) \begin{bmatrix} \phi_S^I \\ \phi_S^{II} \\ \phi_A^I \\ \phi_A^{II} \end{bmatrix}. \quad (10)$$

Next, we move to the more intuitive circularly polarized LCP/RCP basis by applying the following unitary transformation

$$\mathbf{U} = \frac{1}{\sqrt{2i}}\begin{bmatrix} 1 & 0 \\ 0 & 1 \end{bmatrix} \otimes \begin{bmatrix} e^{-i\phi/2} & ie^{-i\phi/2} \\ -e^{i\phi/2} & ie^{i\phi/2} \end{bmatrix} \tag{11}$$

In this basis, the problematic absolute value of $|\delta\mathbf{k}|$ disappears. The phase ϕ is defined as $\delta\mathbf{k} = |\delta\mathbf{k}| \exp i\phi = \delta k_x + i\delta k_y$. Thus, the Hamiltonian ($\mathbf{UHU}^{-1}$) becomes

$$\widehat{\mathcal{H}}_{AS} = \begin{bmatrix} 0 & V_D(\delta k_x - i\delta k_y) & m & 0 \\ V_D(\delta k_x + i\delta k_y) & 0 & 0 & -m \\ m & 0 & 0 & V_D(\delta k_x - i\delta k_y) \\ 0 & -m & V_D(\delta k_x + i\delta k_y) & 0 \end{bmatrix} \tag{12}$$

It is clear that the off-diagonal elements of the matrix induce hybridization between the LCP/RCP modes of the *A* and *S* class of modes. The hybridized modes that are the new unmixed eigenfunctions of the system can be found from the following unitary transformation:

$$\mathbf{U}' = \frac{1}{\sqrt{2}}\begin{bmatrix} 1 & 1 \\ 1 & -1 \end{bmatrix} \otimes \begin{bmatrix} 1 & 0 \\ 0 & 1 \end{bmatrix}. \tag{13}$$

Under this transformation, the new Hamiltonian (obtained by $\mathbf{U}'\mathbf{H}\mathbf{U}'^{-1}$) exactly matches the Kane-Mele low-energy Hamiltonain,[3]

$$\boldsymbol{\mathcal{H}}_{+/-} = \begin{bmatrix} m & V_D(\delta k_x - i\delta k_y) & 0 & 0 \\ V_D(\delta k_x + i\delta k_y) & -m & 0 & 0 \\ 0 & 0 & -m & V_D(\delta k_x - i\delta k_y) \\ 0 & 0 & V_D(\delta k_x + i\delta k_y) & m \end{bmatrix}. \tag{14}$$

This can be written in a compact form using the Pauli matrices:

$$\boldsymbol{\mathcal{H}}_{+/-} = V_D \hat{\tau}_0 \hat{s}_0 \widehat{\boldsymbol{\sigma}}_{\parallel} \cdot \delta\mathbf{k}_{\parallel} + m\hat{\tau}_3 \hat{s}_3 \hat{\sigma}_3 \ , \tag{15}$$

where $\hat{\sigma}_i$, $\hat{\tau}_i$, and $\hat{s}_i$ are the band, inter-valley and pseudo-spin subspace Pauli matrices. Although, we didn't explicitly examine the intervalley subspace (K and K′), the Hamiltonian given in Supplementary Eq. 15 can be directly deduced from the time-reversal symmetry of the system. To put it differently, it is easy to show that the Berry phase of each band around the K point is $\pi \times sgn(m)$ and according to the time-reversal symmetry, the overall Berry phase must be zero, hence, at the K′ point, the mass term should reverse sign.

It is important to note that by reversing the perturbation (carving out the other side of the crystal face instead), the mass term changes sign. This can be easily verified from the unperturbed modes profile and the definition of the Δ_{mn} tensor, given below Supplementary Eq. 8.

Supplementary Note 3 | Topological Character and Spin Chern Number

The dispersion diagram $\omega(\boldsymbol{k})$, historically assumed to contain most of the useful information about the system, lack the information about the system's topology. Topological numbers can only be calculated from the eigenfunctions themselves. Examples of such topological measures are Chern number and Berry phase. Here is the detailed description of how we calculate these numbers for the elastic waves.

Berry connection (or potential) of band *n* is defined as $\vec{\mathbf{A}}^{(n)}(\vec{\mathbf{k}}) = Im\left\{\oint_{unit\ cell} < \vec{\mathbf{u}}^{(\mathrm{n})}(\vec{\mathbf{k}}) \mid \boldsymbol{\nabla}_{\vec{\mathbf{k}}}\vec{\boldsymbol{u}}^{(n)}(\vec{\mathbf{k}}) > dV\right\}$, or in the component representation, it is given by $A_i(\vec{\mathbf{k}}) = Im\left\{\oint_{unit\ cell}\left[u_x^*(\boldsymbol{r})\partial_{k_i}u_x(\boldsymbol{r}) + u_y^*(\boldsymbol{r})\partial_{k_i}u_y(\boldsymbol{r}) + u_z^*(\boldsymbol{r})\partial_{k_i}u_z(\boldsymbol{r})\right]dV\right\}$, where *i* for a 2D crystal can be 1 or 2. Here, $\vec{\mathbf{u}}$ is the displacement field of the eigenmode. The eigenmode amplitude is normalized such that $\oint_{unit\ cell} < \vec{\mathbf{u}}^{(\mathrm{n})}(\vec{\mathbf{k}}) \mid \vec{\boldsymbol{u}}^{(n)}(\vec{\mathbf{k}}) > dV = 1$. To reduce the numerical error, it is best to normalize the arbitrary eigenmode phase by that of an arbitrary (but with non-vanishing magnitude of $\vec{\mathbf{u}}$) point. Also, to calculate the differentiations with enough accuracy, we used a 5-point stencil in each direction.

Berry connection, similar to the electromagnetic vector potential, is gauge dependent. Berry curvature, defined as $\boldsymbol{\nabla} \times \vec{\mathbf{A}}$, similar to the magnetic field, is gauge invariant. In a 2D crystal, Berry curvature is given by $B_z^{(n)}(\vec{\mathbf{k}}) = \partial_{k_x}A_{k_y}^{(n)} - \partial_{k_y}A_{k_x}^{(n)}$. To calculate the Berry curvature, we used again a 5-point stencil in each dimension. Supplementary Figure 4a shows the Berry curvature in the vicinity of the K point for the four bands of interest (shown by circle in Supplementary Figure 3) after the perturbation, shown in Figure 2 of the main text, is introduced. Berry phase, defined as the integration of the Berry curvature, is equal to π ($-\pi$), when the integration is performed near a peak (or a dip). This is similar to the Berry phase of the Dirac bands in integer Quantum Hall effect.

However, this contribution is opposite near the K′ point (Supplementary Figure 4b), dictated by the time reversal symmetry. Hence, the Chern number, given by the integration of the Berry curvature over the entire Brillouin zone (divided by 2π) adds up to zero for Quantum Spin Hall Effect studied in this work. Spin Chern number, defined as the integration of the Berry Curvature of the same spin over the entire Brillouin zone, is, however, nonzero, classifying our synthetic crystal as the first phononic crystal with QSHE. The contribution of each peak (or dip) of the Berry curvature to the Chern number is +1/2 (-1/2). Thus, the Spin Chern number, is $C_S = \pm 1$.

Supplementary Note 4 | Effects of Leakage and Attenuation Caused by Interaction with Viscous Fluid

In the main text, we assumed that the external boundaries of the system are free, that is, when the background environment is vacuum. This is justified due to the significant impedance mismatch between our crystal made of Aluminum and air as the background. Below, we will show that loading of the system with air, or even water, has little effect on the dispersion of the topological elastic modes. We found the interaction with a viscous fluid gives rise to rather marginal effect of dissipation of the bulk and edge modes. By considering realistic values for the fluid viscosity, it was found that the lifetime of the modes (in the unit of the elastic wave period) exceed the value of **10,000** in the spectral range considered (~130 kHz). Since the viscosity scales as the frequency squared, this results in a greater impedance mismatch for higher frequencies. One therefore may expect that the modes lifetimes would be even higher at higher frequencies. Nevertheless, the topological nature of the modes remains intact by these loss channels, which also agrees with recent studies of topological states in open and dissipative condensed matter systems[4].

The acoustic pressure field p in a homogenous medium satisfies the wave equation[5–7], $(k_0^2 + \nabla^2)p = 0$, where $k_0 = \omega/c - i\alpha(\omega)$. Here, ω is the angular frequency, and c is the speed of sound in the medium, which is proportional to the fluid density ρ, and $\alpha(\omega)$ is the attenuation, caused by the viscosity and relaxation mechanisms in the viscous fluid. Attenuation in air is assumed to be $1.42 \times 10^{-11} f^2\ \mathrm{Np.m^{-1}}$ as a function of frequency f (Ref. 5, page 302 and 306), which gives the value α =0.24 $\mathrm{Np.m^{-1}}$ at 130 kHz. For water, this number is much lower 0.00092 $\mathrm{Np.m^{-1}}$ (or 8 dB/km).

The interaction of the elastic wave with the viscous fluid is implemented by subjecting the boundaries between the fluid and elastic media to the following boundary conditions: 1) the continuity of the acceleration along the normal direction: $-\frac{1}{\rho}\nabla p \cdot \vec{\mathbf{n}} = \frac{d^2}{dt^2}\,\vec{\mathbf{u}} \cdot \vec{\mathbf{n}}$, where $\vec{\mathbf{n}}$ is the unit normal vector, and 2) the elastic traction on the boundary, instead of vanishing for a free boundary case, equals the fluid pressure $\mathbf{T} \cdot \vec{\mathbf{n}} = p\vec{\mathbf{n}}$.

First, we investigate the effect of fluid interaction on the bulk modes of our metacrystal. Supplementary Figure 7 shows the modified dispersion diagram of the bulk propagating modes due to the presence of air and water. The lifetime of the modes, defined as the ratio of the real part to the imaginary part of the eigenfrequency, is also plotted by color in log scale. As can be seen from Supplementary Figure 7a-b, when compared to the solid curves (corresponding to the ‘free boundary’ case), the dispersion diagrams are barely modified. The only change is that the quality factors acquire finite, but still very large values. We also investigated the role of loss at different frequencies by down scaling the structure. The results presented in

Supplementary Figure 8 show that role of loss diminishes as the fourth power of frequency and becomes barely noticeable when entering the MHz frequency range.

Finally, we study the influence of the viscous fluid on the topologically robust edge modes. As can be seen from Supplementary Figure 9, the effect of water loading on the edge modes is only a change in their lifetime; their dispersion is barely altered. Nonetheless, the edge modes can propagate for tens of thousands of unit cells before dissipating away.

Supplementary Note 5 | Surface Modes of Topologically Trivial Crystal

As shown in shown in Fig. 4 of the main text, a line defect may be created by half-filling one of the rows in the topologically trivial crystal, given in Fig. 1 of the main text. Such a line defect supports topologically trivial surface modes, as shown by the red curves in Supplementary Fig. 5. These modes are topologically trivial as they do not connect the top and bottom propagation bands.

Supplementary Note 6 | Topologically Trivial Surface Modes of Free and Clamped External Boundaries

The domain walls between two topologically nontrivial crystals with opposite mass term signs support topologically robust edge modes. Investigation of the field profiles shows that there is a finite stress tensor (particularly its normal components) on the domain wall for all four edge modes. Indeed, removing one of the domains on either side of the wall renders the now external boundary stress free ($\hat{T} \cdot \boldsymbol{n} = 0$), hence, the boundary condition is not satisfied for the appearance of the same topologically robust edge modes. Left panel on Supplementary Figure 6 shows the edge modes residing on such free external edges. Similar outcome occurs for a clamped ($\boldsymbol{u} = 0$) boundary (shown in the right panel). These surface modes whose dispersions are gapped are topologically trivial.

Supplementary References

1. Royer, D. & Dieulesaint, E. *Elastic Waves in Solids I: Free and Guided Propagation*. (Springer Science & Business Media, 2000).
2. Slater, J. C. Microwave Electronics. *Rev. Mod. Phys.* **18,** 441–512 (1946).
3. Kane, C. L. & Mele, E. J. Quantum Spin Hall Effect in Graphene. *Phys. Rev. Lett.* **95,** 226801 (2005).
4. Baum, Y., Posske, T., Fulga, I. C., Trauzettel, B. & Stern, A. Coexisting Edge States and Gapless Bulk in Topological States of Matter. *Phys. Rev. Lett.* **114,** 136801 (2015).
5. Blackstock, D. T. *Fundamentals of Physical Acoustics*. (Wiley-Interscience, 2000).
6. Kinsler, L. E., Frey, A. R., Coppens, A. B. & Sanders, J. V. *Fundamentals of Acoustics*. (Wiley, 1999).
7. Reyes-Ayona, E., Torrent, D. & Sánchez-Dehesa, J. Homogenization theory for periodic distributions of elastic cylinders embedded in a viscous fluid. *J. Acoust. Soc. Am.* **132,** 2896–2908 (2012).
