# Supplementary figures and images for: Topologically protected elastic waves in phononic metamaterials
Source: Nat Commun. 2015 Nov 4;6:8682. doi: 10.1038/ncomms9682 (PMC4659837; doi:10.1038/ncomms9682)

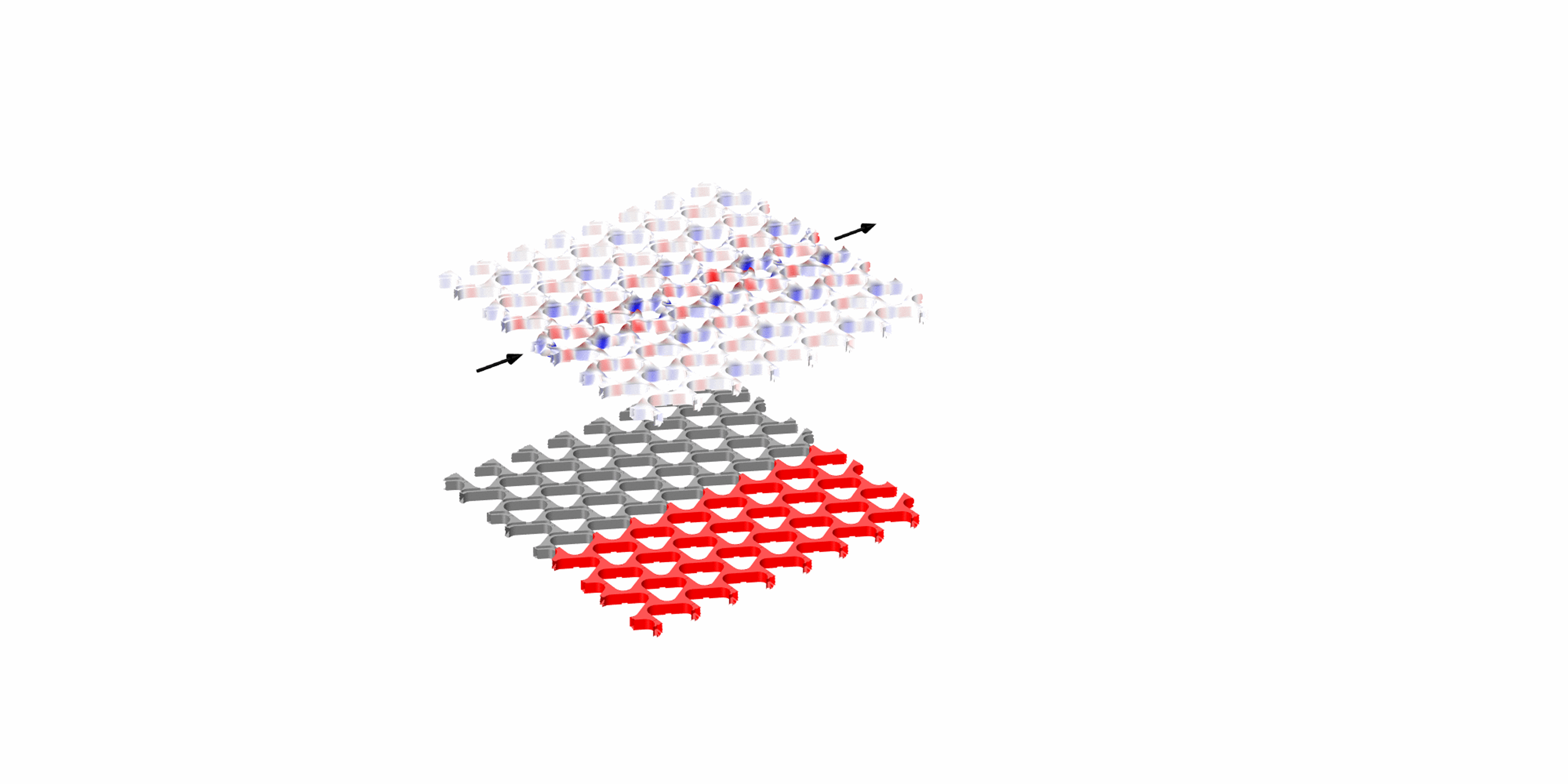

Supplement: Supplementary Movie 1 — The flow of the forward-propagating edge mode on the domain wall between two topologically nontrivial crystals with opposite mass terms. The arrows show the input and output ports. [file ncomms9682-s2.tif]

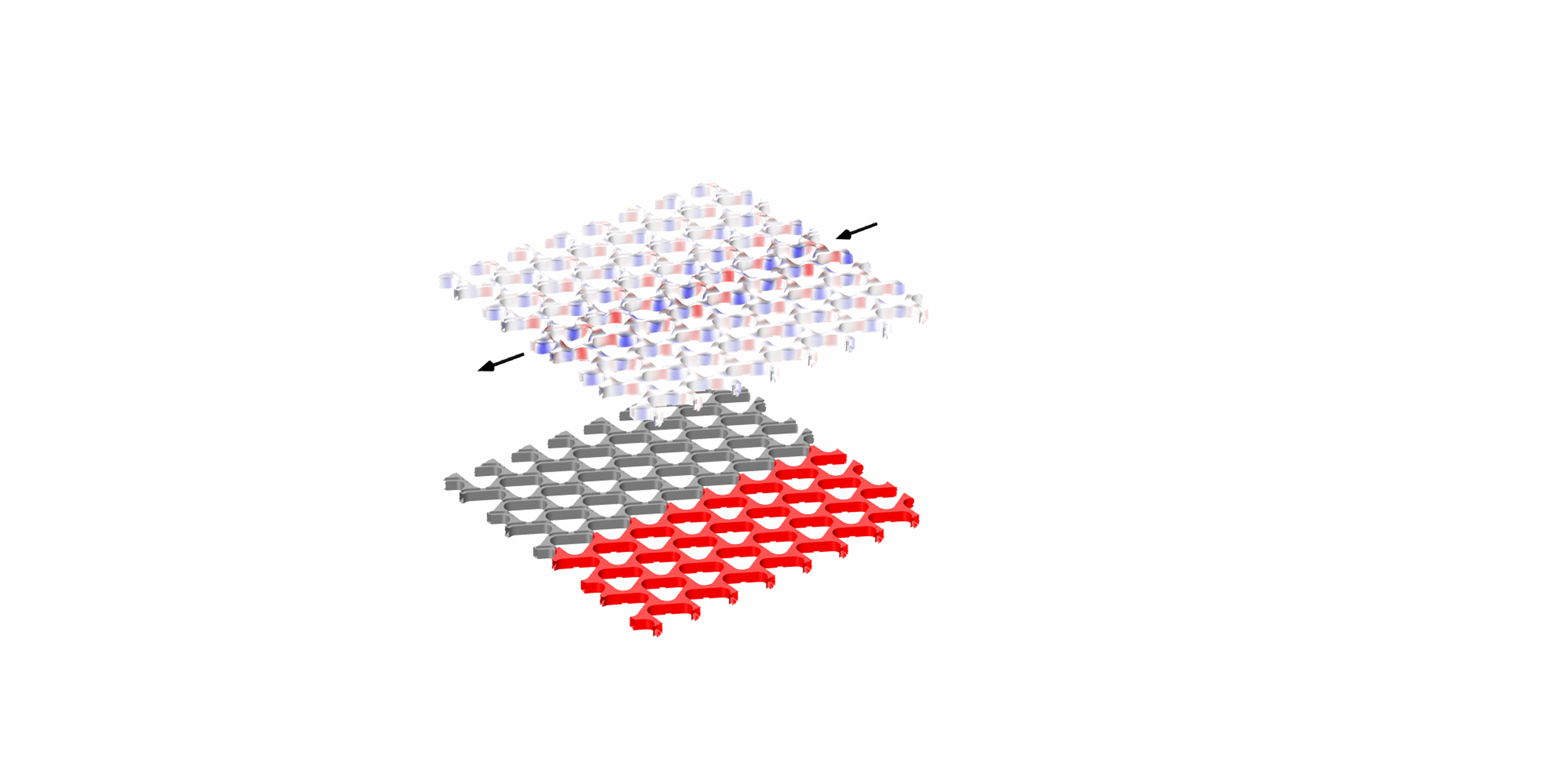

Supplement: Supplementary Movie 2 — The flow of the backward-propagating edge mode on the domain wall between two topologically nontrivial crystals with opposite mass terms. The arrows show the input and output ports. [file ncomms9682-s3.tif]
